# Circulating miRNAs Related to Epithelial–Mesenchymal Transitions (EMT) as the New Molecular Markers in Endometriosis

**DOI:** 10.3390/cimb43020064

**Published:** 2021-08-05

**Authors:** Anna Zubrzycka, Monika Migdalska-Sęk, Sławomir Jędrzejczyk, Ewa Brzeziańska-Lasota

**Affiliations:** 1 Department of Biomedicine and Genetics, Medical University of Lodz, Pomorska 251, C-5, 92-213 Lodz, Poland; monika.migdalska-sek@umed.lodz.pl (M.M.-S.); ewa.brzezianska@umed.lodz.pl (E.B.-L.); 2 Operative and Conservative Gynecology Ward, Dr. K. Jonscher Municipal Medical Centre, Milionowa 14, 93-113 Lodz, Poland; slawekjedrzej@interia.pl

**Keywords:** endometriosis, microRNAs, epithelial–mesenchymal transitions (EMT), non-invasive biomarker

## Abstract

Endometriosis is a chronic gynecological disease defined by the presence of endometrial-like tissue found outside the uterus, most commonly in the peritoneal cavity. Endometriosis lesions are heterogenous but usually contain endometrial stromal cells and epithelial glands, immune cell infiltrates and are vascularized and innervated by nerves. The complex etiopathogenesis and heterogenity of the clinical symptoms, as well as the lack of a specific non-invasive diagnostic biomarkers, underline the need for more advanced diagnostic tools. Unfortunately, the contribution of environmental, hormonal and immunological factors in the disease etiology is insufficient, and the contribution of genetic/epigenetic factors is still fragmentary. Therefore, there is a need for more focused study on the molecular mechanisms of endometriosis and non-invasive diagnostic monitoring systems. MicroRNAs (miRNAs) demonstrate high stability and tissue specificity and play a significant role in modulating a range of molecular pathways, and hence may be suitable diagnostic biomarkers for the origin and development of endometriosis. Of these, the most frequently studied are those related to endometriosis, including those involved in epithelial–mesenchymal transition (EMT), whose expression is altered in plasma or endometriotic lesion biopsies; however, the results are ambiguous. Specific miRNAs expressed in endometriosis may serve as diagnostics markers with prognostic value, and they have been proposed as molecular targets for treatment. The aim of this review is to present selected miRNAs associated with EMT known to have experimentally confirmed significance, and discuss their utility as biomarkers in endometriosis.

## 1. Introduction

Endometriosis is an estrogen-dependent chronic gynecological disease with mixed features of benign disease and malignancy. Although spontaneous remission is possible, it is suggested that endometriosis may progress to carcinogenesis [1]. It is characterized by the presence of endometrial tissue and the growth of dysfunctional endometrial glands and stroma outside the uterine cavity [2]. The typical clinical symptoms are dysmenorrhea, dyspareunia, dyschezia, dysuria, infertility, cyclic and acyclic pelvic pain [3,4]. Despite the severity of some of these symptoms, as many as 11% of cases go undiagnosed [5], and symptomatic endometriosis occurs in approximately 10% of all women of reproductive age [6,7]. A recent meta-analysis found the total incidence of endometriosis, to demonstrate high heterogeneity, ranging from 1.36–3.53/per 1000 person years depending on the source of information [8].

Limited evidence suggests that the incidence of endometriosis can also depend on population type and ethnicity, with black women being less frequently diagnosed with endometriosis, and Asian women more likely, than white women. Moreover, there was a significant difference in the likelihood of endometriosis diagnosis between Hispanic and white women [9]. However, is not clear whether this variation reflects differences in response to treatment among ethnic groups [10].

A number of other interrelated risk factors for endometriosis have been identified, including clinical characteristics, hormonal, immunological, genetic, epigenetic, and environmental factors. The development and progression of the disease may also be associated with certain familial predispositions [11,12]. The fact that the endometriosis phenotype demonstrates such variability suggests that a single etiopathogenetic explanatory model is not sufficient. Indeed, there are many inconsistent theories and hypothesis concerning the pathogenesis of endometriosis [11,12,13]. One of the most popular, associated with intraperitoneal and ovarian endometriosis, is Sampson’s implantation theory, or the retrograde menstruation theory, which proposes that eutopic fragments of the endometrium are the implanted outside the uterine cavity as a result of menstrual reflux through the fallopian tubes [14]. Ectopic tissue implantation and proliferation is facilitated by an inflammatory process that escapes immune surveillance, which has been suggested to be overloaded or insufficient in women with endometriosis [7,15].

A recent theory concerns the role of M1/M2 macrophage activity and its polarization in endometriosis, with the transition from classical M1 macrophage activity to alternative M2; this correlates with the histological features of the initial acute inflammation and subsequent with pro-fibrotic activity and the process of tissue remodeling characteristic of advanced stages of endometriosis. In fact, M1 macrophages have been found to be more abundant in ovarian endometriosis in stages I–II, while M2 macrophages are found in large amounts in stages III-IV [16]. It has been found that peritoneal macrophages demonstrate lower cytotoxic potency in the peritoneal cavity of women with endometriosis and the pattern of secreted cytokines and chemokines is altered. However, it has not been clarified whether the change in the peritoneal microenvironment is a cause or a consequence of endometriosis [7].

As such, many alternative hypotheses exist for the development and dissemination of endometrial implants, especially those remote from the peritoneal cavity. Some authors propose that the main feature of endometriosis is bone marrow-derived stem cell trafficking. Due to their contribution to unlimited cell proliferation and to a high developmental plasticity, they are able to differentiate directly into endometriotic cells at ectopic locations and infiltrate the eutopic endometrium [17,18,19]. Emerging evidence suggests that the endometriotic phenotype of stem cells may be modulated by microRNA. Significant epigenetic changes in the level of miRNA expression in turn result in dysregulated expression of their target genes involved in epithelial–mesenchymal transition (EMT), which can be associated with the proliferation, migration and the local invasion of the endometrial cells at ectopic sites [17,20,21,22]. This review highlights the importance of selected circulating EMT-related miRNAs in the pathophysiology of endometriosis and examines their potential as diagnostic biomarkers for endometriosis.

## 2. Key EMT Regulators Related to Endometriosis

The phenomenon of epithelial–mesenchymal transition (EMT) has long been associated with endometriosis, although there is little research confirming the role of EMT in the etiopathogenesis of the latter [11,12]. The transformation process is characterized by a loss of polarity and cell-to-cell contacts within epithelial cells and mesenchymal cells gaining the ability to migrate and invade other regions. Moreover, mesothelial cells undergoing EMT transformation lose the ability to act as a protective barrier between the basal layer and the luminal space. Both mesothelial and epithelial-to-mesenchymal transition seem to be key in endometriosis development. These abilities are also thought to be fundamental for the establishment of endometriotic ectopic lesions [21]. Moreover, a main feature of EMT is anoikis resistance, an attachment-free cell survival which could play an important role in the spread of ectopic lesions through the promotion of a pro-survival signal [23].

Different biological types of EMT (1–3) are believed be involved in the development of endometriosis lesions. Type 1 occurs during embryogenesis and is not associated with inflammation, fibrosis or metastatic process [24,25]. In contrast, type 2 EMT is a repair-associated process that is normally involved in creating fibroblasts and other related cells in order to reconstruct tissues following trauma and inflammatory injury. As such, type 2 EMT is associated with persistent inflammation, fibrosis, wound healing and regeneration [25]. Type 3 EMTs is believed to be a key cause of tumor metastasis. It occurs in neoplastic cells displaying genetic and epigenetic alterations. Finally, molecular changes may disturb gene function, particularly that of pro-oncogenic and tumor suppressor genes.

It has recently been proposed that type 2 EMT, and type 3 to a lesser extent, might be involved in the pathogenesis of this disease [26]. Moreover, endometriosis has been recognized as an inflammatory disease, demonstrating immune system dysfunction [27]. It has been proposed that Type 2 EMT might be involved in a range of pathogenic processes involved in endometriosis development, such as immunological response dysfunction, inflammation, chronic inflammation and hypoxia injury fibrosis [28,29]. Few reports have examined the inflammatory pathways involved in the progression of endometriosis and which may induce synergistic elevation of inflammatory process. It has been found that the Toll-like receptor (TLR)-dependent signaling pathway, probably via endogenous ligands, may result in increased microbial stimuli and contribute to microbial-related or sterile inflammation in endometriosis. In particular, upregulation of pro-inflammatory pathways through endogenous ligand/TLR signaling combined with activation of nuclear factor-kB (NF-kB) signaling in response to oxidative stress, may play a significant role [6,30].

Several review articles have emphasized the role of NF-kB activation in the development of endometrial lesions [31,32]. In addition, activation of NF-kB transcription factors is believed to be dependent on increased production of reactive oxygen species (ROS), which are able to stimulate mitogen activated protein kinase (ERK/MAPK) pathways [32,33,34,35]. It has been shown that immune response disorders, mostly type 2 EMT, observed in the microenvironment, slow down the progression of endometrial changes, probably by suppressing transforming growth factor-β1/Smad3 (TGF-β1/Smad3) signaling pathways [36]. It is known that TGF-betas and their high-affinity receptors are abundantly and differentially expressed in the endometrium under hormonal control; as such, their increased expression may be essential for the establishment and/or maintenance of endometriosis [37]. Moreover, several cytokines, including the two strongest stimuli of fibrogenesis, platelet-derived growth factor (PDGF) and TGF-β1, known to promote EMT and fibroblast-to-myofibroblast transition, can induce increased collagen production, cell contractility and smooth muscle metaplasia in lesions, eventually resulting in fibrosis. These processes have often been found in peritoneal, ovarian, extragenital and pleuropulmonary endometriosis [38,39,40,41]. The aberrant interaction of the TGF-β/Smad and Wnt/β-catenin pathways in mediating fibrogenesis in endometriosis is also emphasized [42,43,44]. Other studies suggest that activation of the Wnt/β-catenin signaling pathway may promote EMT [45,46].

TGF-β not only promotes cell proliferation in ovarian endometrial cysts but could activate the expression of vascular endothelial growth factors (VEGF-s) in the peritoneal mesothelial cell to support endometrial lesion vascularization [47,48]; in addition, dysregulation of hypoxia induced factor (HIF)-1α and matrix metallopeptidase (MMP) is also believed to play a role [49]. Initiation of EMT3 and the enhanced metastasis ability of mesenchymal/mesothelial cells observed during the progression of endometriosis is often linked to angiogenesis, which promotes endothelial cell dysfunction and invasions by ectopic lesions. The cascade of EMT3-related molecular processes involved in the progression of endometriosis has been confirmed in an animal model. In particular, vascular endothelial growth factor C (VEGF-C) plays an important role in the angiogenesis mechanism and also serves as a potential therapeutic target of anti-angiogenesis for endometriosis [50].

Additionally, increased VEGF secretion was observed in hypoxia-induced endometrial stromal and glandular cells compared with that under normoxic conditions [51,52]. More interestingly, Lin et al. report that hypoxia increases cell adhesion, and that HIF-1α stabilization could enhance the level of SMAD2/SMAD3 phosphorylation in primary endometrial stromal cells [53,54]. Hypoxia is regarded as the upstream regulator that increases the invasiveness of endometriotic cells [55]. The imbalance between the levels of MMPs and tissue inhibitor of metalloproteinase (TIMP), as well as disorders in their modulators, have been found to influence the regulation of cellular migration and invasion in endometriosis [56,57,58].

**Figure 1:**
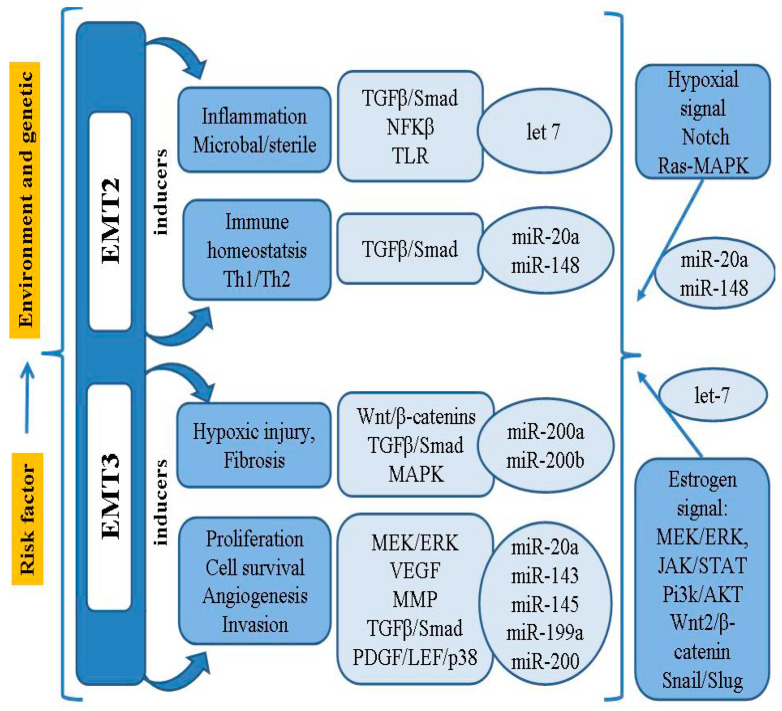
Summary model for the pathogenesis and risk factor of EMT-related endometriosis.

In addition to the MMPs involved in the cellular event of EMT, it has been found that the activity of Snail, Slug and Twist, transcriptional factors binding to the E-cadherin promoter, may also be associated with loss of differentiation, progression and metastasis [21]. It has been confirmed that the Notch signaling pathway can inhibit the development of EMT, as well as ectopic endometrial stromal cell migration and invasion, by down-regulating Snail, Slug and Twist [21,59]. Thus, the process of EMT in endometriosis requires a series of complex changes in cell architecture and behavior; these are driven by a range of cellular signals that influence genetic/epigenetic signatures, as well as the microRNA (miRNAs/miR) profiles closely connected with the regulatory network of genes. A summary model of the pathogenesis and risk factors of EMT-related endometriosis is shown in Figure 1.

## 3. Role of miRNAs in the Pathophysiology of Endometriosis

The diagnosis of endometriosis can only be established by direct visualization of the lesions during invasive laparoscopic surgery, ideally with histological confirmation [60,61]. Different imaging techniques, such as ultrasound, computed tomography and magnetic resonance imaging, have proven unreliable in the diagnosis or staging of the disease [62]. Therefore, there is a need for a search for a reliable, highly specific, non-invasive test that can be used in both cases [3].

The recent discovery of miRNAs as stable and specific modulators of gene expression has enabled their use in numerous diseases as diagnostic and prognostic biomarkers. Various studies of miRNAs have identified their important role in the pathogenesis and diagnosis of endometriosis. At the endometrial level, miRNA is involved in the dynamic changes associated with the menstrual cycle and the pathophysiology of reproductive disorders, such as endometriosis and recurrent miscarriages [63].

MicroRNAs are small molecules of ribonucleic acid (RNA) which act as post-transcriptional gene expression regulators. They repress protein synthesis by inhibiting the translation of the target messenger RNA (mRNA). They play essential roles in the epigenetic control mechanisms of many biological and physiopathological processes, such as proliferation, differentiation, migration, matrix remodeling, angiogenesis, and apoptosis [64,65]. It is currently known that miRNA can regulate many mRNAs, and at the same time one mRNA can be regulated by various miRNAs [66,67]. They are one of the most important molecules influencing the information pathways in cells. miRNA molecules are present both intracellularly and in the inter/extracellular space, as well as in circulating bodily fluids (bio-fluids). miRNAs can be transported into the systemic circulation in exosomes or microvesicles, in which they can be incorporated into distant cells with functional consequences [68,69]. Moreover, it has been shown that miRNAs circulate in the blood stream under the protection of high-density lipoprotein (HDL), and are delivered, with functional targeting capabilities, to recipient cells [70]. It is known that aberrant miRNA expression usually causes dysregulation of the target genes involved in the development of many diseases, including endometriosis [71,72].

Numerous studies have confirmed that the miRNA expression profile is altered in both eutopic and ectopic endometrial tissues in women with endometriosis compared to controls [63,71,72,73,74,75]. Four miRNAs, viz. miR-34c-5p, miR-9-3p, miR-9-5p and miR-34b, were found to be downregulated in eutopic endometrial tissues of women with endometriosis [76], while the most frequently deregulated miRNAs in endometriosis are believed to be miR-200, miR-143, miR-145, miR-20a, miR199a and the let-7 families [22,77]. In addition, an miRNA candidate panel comprising miR-20a-5p, miR-199a-3p, miR-143-3p and let-7b-5p showed diagnostic potential in differentiating healthy women from women with endometriosis with a similar sensitivity and specificity to laparoscopy [73,78].

Liquid biopsy in the form of plasma, serum, whole blood, or peritoneal fluid supernatant has been found to demonstrate an altered miRNA signature patients with endometriosis [79]. The samples from patients demonstrate increased expression of numerous miRNAs in blood serum (miR-125b-5p, miR-150-5p, miR-342-3p and miR-451a) [80] or in serum exosomes (miR-22-3p and miR-320a, miR-197-5p, miR-320b, miR-3692-5p, miR-4476, miR-4530, miR-4532, miR-4721, miR-4758-5p, miR-494-3p, miR-6126, miR-6734-5p, miR-6776-5p, miR-6780b-5p, miR-6785-5p, miR-6791-5p, miR-939-5p) [81] or in plasma (miR-145, miR-33a-5p) compared to controls [82,83]. Other miRNAs have been found to be downregulated in serum samples (miR-3613-5p and let-7b, miR-6313, miR-142-3p, miR-17) or in serum exosomes (miR-134-5p, miR-3141, miR-4499, miR-6088, miR-6165, miR-6728-5p [84], or in plasma (miR-154-5p, miR-196b-5p, miR-378a-3p) [83] from patients with endometriosis compared to controls [80,85,86].

In addition, elevated levels of CD14 + monocytes/macrophages and miRNAs have been reported in peritoneal fluid supernatant from endometriosis patients. For example, miR-146b can inhibit M1 polarization of endometrial stromal cells co-cultured with macrophages [87], which are reported to be the main source of pro-inflammatory chemotactic cytokines and the main source of neuroangiogenesis [88]. The evolution of endometriosis has been linked to a range of M1 macrophage polarization markers, including tumor necrosis factor α (TNF-α)—a marker with a strong inflammatory, cytotoxic and angiogenic potential, and IL-1β, IL-12, IL-8, IL-10 and IL-6, which promote the growth of endometrial cells. Importantly, the levels of secreted inflammatory cytokines of macrophages has been found to correlate with changes in miR-125b-5p and let-7b-5p in the serum of patients with endometriosis [89]. Other miR-146 targets, such as IRAK1, TRAF6, STAT1 and IRF5, play a key role in mediating M1 polarization; hence their regulation in endometrial tissue may be important in the etiopathogenesis of endometriosis [87].

The mechanism by which endometriosis systemically alters macrophage cytokine production may be based on the activity of miRNAs, which, when transported in the exosomes, can regulate genes in distant cells. For example, miR-22-3p was found to be significantly increased in exosomes derived from peritoneal macrophages from endometriosis; it appears to be delivered from macrophages to ectopic endometrial stromal cells via exosomes, where it promotes the proliferation, migration, and invasion cells by targeting SIRT1 and activating the NF-κB pathway [84]. Of particular interest may be miR-451, which is believed to target the macrophage migration inhibitory factor (MIF). This mitogenic cytokine can shape a proliferative and angiogenic phenotype conducive to the establishment and/or growth of endometriosis. In addition to the epithelium of endometrial lesions [90] and peritoneal macrophages [91], levels of MIF are also elevated in peripheral blood [92] and in peritoneal fluid [93,94] in women with endometriosis. The relative expression of MIF was negatively correlated with that of miR-451, and it is possible that the modulation of this miRNA expression may underpin the mechanism limiting the survival of endometriotic lesions [95]. Apart from the aforementioned miR-451a, which is suggested to be a mediator of inflammation and T-cell immunity [96], higher expression in the peritoneal cavity of patients with endometriosis is also found in miR-106b-3p and miR-486-5p [97]. In addition, the increase in the level of miR451a and miR-486-5p positively correlates with the severity of the disease, and exosomal miRNA-1908, -130b, -4488, -432, -342, -425, -505, -6508, -145, -365a and -365b were differently expressed in both early and advanced stages of endometriosis compared to the control group [98]. Two of the latter, i.e., miRNA-1908 and -130b, have been proven alter immune cells such as regulatory T cells and macrophages [99,100] and, together with miRNA-451a, may contribute to the abnormal peritoneal immune microenvironment in patients with endometriosis [98]. Peritoneal cytokine profile in infertile patients with endometriosis was related to increased levels of stem cell growth factor b, hepatocyte growth factor (HGF), monocyte chemoattractant protein 1 (MCP-1) and IL-8, while IL-13 decreased significantly [101]. Mention should also be made of other cytokines, inflammatory and adhesive factors which, as messengers, can influence the immune cell response and contribute to the evolution of endometriosis. These are: CXCL1, CXCL2 and MCP-2/3, TNF-α, TNFR1, fibroblast growth factor 2, vascular cell adhesion molecule, αV and β3 integrins, interleukin 1 α (IL-1α) and macrophage inflammatory protein 1b [98]. Thus, in the light of the above data, the exosomal miRNA is an important factor changing the immune microenvironment in the cavity of the peritoneum of women with endometriosis.

On the one hand, circulating miRNAs have become attractive biomarkers in endometriosis due to their lower complexity, tissue specificity and stability in urine, blood and tissues [102]. On the other hand, as the level of miRNA expression depends on the phase of the menstrual cycle, hormonal dysregulation and circadian variability may result in differentiated expression [74,76,103]. In addition, studies have been based on a range of different test technologies and cell types. Nevertheless, miRNA research opens up new horizons in understanding the pathogenetic molecular events of endometriosis, with the aim of determining the potential of circulating miRNAs as biomarkers of endometriosis. However, more research is still needed as no single or panel miRNAs seem to meet the criteria for a diagnostic biomarker in endometriosis.

## 4. Candidates of Circulating miRNAs Related to EMT in Endometriosis

### 4.1. miR-200

The miR200 family members (miR-200s) consists of miR-200a, miR-200b, miR-200c, miR-141 and miR-429 [104]. Ohlsson Teague et al. (2009) report that the combination of three of the most studied miRNAs associated with endometriosis (e.g., miR-200a, miR-200b and miR-141) had a sensitivity and a specificity of 84.4% and 66.7%, respectively [105]. Ohlsson Teague et al. (2009) and Hawkins (2011) indicate that miR-200 family were down-regulated in the ectopic endometrium compared to the eutopic endometrial tissue [105,106]. It has been proven that a significant down-regulation in the miR-200 family may be linked to the acquisition of a migratory mesenchymal phenotype characteristic of the EMT process, during which epithelial cells lose their specific features and integrity, and acquire mesenchymal characteristics, leading to increased cell invasion and migration [26,107]. Furthermore, based on in vivo data, it is hypothesized that EMT miR-200b may have a repressive function on endometriotic cells, although it is dependent on the tissue and the expression of factors in the microenvironment [17]. Possibly due to up-regulation of the pluripotency-associated transcription factor KLF4 [17], miR-200b has also been shown to play a role in modulating proliferation and differentiation of stem cells [104].

The miR-200 family members target a complex network of transcription regulators like (*ZEB1* and *ZEB2*), which are transcriptional repressors for E-cadherin. The overexpression of miR-200 family members results in decreased expression of ZEB1/ZEB2 and increased expression of E-cadherin, which is required for maintaining the epithelial nature of cells [76,108]. miR-200 family members block EMT by inhibiting ZEB1 and ZEB2 expression and by targeting metastasis associated lung adenocarcinoma transcript 1 (MALAT1) in endometriosis [109]. MALAT1 upregulation and miR200 downregulation have been observed in endometriosis [106,109]. Most recently, it has been noted that E2-mediated MALAT1/miR200s axis may affect cell migration and invasion in endometriosis by regulating EMT, and hence may represent a potential therapeutic target [110].

It has been proposed that of the miR200 family, MiR200c demonstrates the strongest interaction with MALAT1. In addition to miR-429, the expression of miR-200a, miR-200b, miR-200c and miR-141 has also been found to be downregulated in endometriosis [64,105,106,111,112,113]; some of them, e.g., miR-200b and miR-200c, also appear to be negatively correlated with *VEGF* expression in endometriotic tissues. This has been associated with higher proteolytic and angiogenic activities and, consequently, easier cell implantation in eutopic regions [113].

Rekker et al. (2015) studied the serum expression of the miR-200a-3p, miR-200b-3p, and miR-141-3p [74]. They concluded that the expression of all these members was down-regulated in patients compared to controls, and that miR-200a-3p and miR-141-3p had the highest potential as noninvasive biomarkers for endometriosis. miR-141 has been identified as a novel driver of EMT in endometriosis, with a possible link existing between miR-141 and the TGF-β1/SMAD2 signaling pathway in the context of endometriosis, and underscoring the role of EMT in the development of endometriosis [114]. In addition, EMT is associated with a decrease in miR-141–5p and an increase in Notch-1/Hes-1 expression in endometriosis [115].

Interestingly, miR200s panel analysis has also been found to yield different results depending on the time of the day at which blood collection occurred, with lower levels of circulating miRNAs being recorded in the evening. Hence, the impact of circadian rhythms on serum miRNA levels may also play a role in the inconsistency between studies [74].

### 4.2. miR-20a

Another circulating miRNA considered to be a leading biomarker for endometriosis is miR-20a. MiR-20a is a part of the miR-17-92 cluster, which encodes a miRNA polycistron that yields six miRNAs (miR-17, miR-18a, miR-19a, miR-19b, miR-20a and miR-92a) [77]. It has been shown to be downregulated [73,78,105,111,116,117], or upregulated [118,119] in plasma and endometriotic lesion biopsies from patients with endometriosis. Stratified analysis showed miR-20a to play a pivotal role in the etiopathogenesis of ovarian endometriosis by influencing cell cycle progression, and that while increased miR-20a expression was associated with advanced endometriosis (stage III-IV), no such relationship was observed for mild endometriosis (stage I-II) [119]. Abnormal circulating miRNA levels result in the cell becoming insensitive to the signals directing it to the apoptosis pathway. This happens in the case of miR-20a, where a low level of miR-20a expression regulates the translation of B-cell lymphoma 2 (BCL2), which codes for an anti-apoptotic protein, and cyclin-dependent kinase inhibitor 1A/p21 (CDKN1A/p21), a cell cycle repressor. Moreover, miR-20a targets E2F transcription factor 3 (E2F3) and Cyclin D1 (CCND1), and thus participates in epithelial cell proliferation and decreased apoptosis (antiapoptotic action); it also targets interleukin-8 (IL-8) and TGF-β, which promotes epithelial–mesenchymal transition in endometriosis. Thus, down-regulation of miR-20a leads to increased concentrations of pro-inflammatory molecules, which in turn, promote an inflammatory milieu and tissue repair, thus contributing to the growth of endometriotic lesions [77]. Down regulation of miR-17-5p/20a is also associated with overexpression of hypoxia-inducible transcription factors (HIF-1a) [120] and pro-angiogenic vascular endothelial growth factor (VEGF-A) [121], leading to neoangiogenesis [122]. miR-20a also downregulates anti-angiogenic thrombospondin 1 (TSP-1) and promotes neovascularization, which is necessary for the implantation of ectopic foci [116]. miR-20a is also known to act as a hypoxamir, and its overexpression in endometriotic stromal cells is a critical factor for the downregulation of the dual-specificity phosphatase-2 (DUSP2) and increase in the expression of ERK target genes related to angiogenesis: (early growth response protein-1; *EGR-1*, cysteine-rich angiogenic inducer 61; *CYR61*, osteopontin) and proliferation (prostaglandin-E2; *PGE2*, cyclooxygenase-2; *COX-2*, and fibroblast growth factor; *FGF-9*) [118]. Interestingly, FGF-9, as an autocrined estromedin, can control an important step in the development/maintenance of endometriosis in normal endometrium. It is a potent mitogen for endometrial stromal cells, and its over-expression found in the early stage of endometriosis may improve the survival of ectopic endometrial lesions by encouraging rapid cell proliferation [123].

To summarize, miR-20 is an important modulator of a number of processes related to the etiopathogenesis of endometriosis, including promoting the growth of endometrial and endothelial cells, proliferation and angiogenesis [77,116,118,122]. However, further research is needed before miR-20 can be used as a biomarker in the diagnosis of endometriosis.

### 4.3. miR-199a

The expression of miR-199a is highly heterogeneous between studies, which may result from the differences in the analysis methods, patient group size or biological material used in research projects. A comparison of miR-199a levels indicated lower expression in ectopic than in eutopic endometrial stromal cells from the same patient [124]. miR-199a expression has been found to be much lower in eutopic endometrial tissue from women with endometriosis compared with normal controls, and even lower in paired ovarian endometrioma [124,125,126]. It was therefore suggested that miR-199a may play a role in the initiation of endometriosis [125]. Indeed, miR-199a was significantly downregulated in blood samples from patients with endometriosis [124], although interestingly, miR-199 showed a remarkably high expression in recurring cases [116]. Some studies indicate that serum miR-199a has 100% sensitivity and specificity in diagnosing endometriosis, and therefore may be a suitable biomarker for endometriosis [127]. However, another study found no differences in miR-199a levels between endometriomas and endometrium [106].

Despite their differential expression, miR-199a has been shown to be functionally linked to hormone-mediated signaling pathways. Under hypoxic conditions, which may be an early phenomenon in endometrial tissue transplantation and a key factor in endometrial angiogenesis, miR-199a blocks the angiogenic potential of stromal cells by targeting two essential genes, *VEGF-A* and *HIF1A*, in the HIF-1α/VEGF-A signaling pathway [128]. Another study showed that miR-199a decreased VEGFA expression by directly targeting the 3’ UTR of its mRNA, resulting in inhibition of proliferation, motility and angiogenesis in ectopic endometrial mesenchymal stem cells. These researchers also found that miR-199a-5p could decrease the size of endometriotic lesions in vivo, and might facilitate the development of potential therapeutics against endometriosis; it is possible that this miRNA may be involved in the mechanism of action of anti-estrogen drugs like danazol [124]. It has been speculated that miR-199a-5p might target IκB-β kinase (IKBKB), which further activates NF-κB and regulate IL-8 secretion. Therefore, miR-199a inhibition would increase the expression of these inflammatory mediators, thus increasing the invasive capability of endometrial cells, and decreasing endometrial receptivity and implantation defects [125]. Wang et al. (2013) report the presence of higher levels of miR-199a in the serum of women with endometriosis, suggesting that it could potentially serve as a non-invasive biomarker for endometriosis. They propose that the upregulation of miR-199a in endometriosis regulates adhesion and produces a deep infiltration of endometrial cells by targeting three genes: *CLIC4*, *RTN4* and *VCL* [75].

The studies discussed above suggest that miR-199a may play an important role as regulator of endometriosis progression and make a contribution to its treatment. This effect is confirmed by a recent study which showed that miR-199a-5p targeted ZEB1 to inhibit the EMT of ovarian ectopic endometrial stromal cells via the PI3K/Akt/mTOR signal pathway. This axis also appears to be overactive in endometrial pathologies and promotes the survival and proliferation of diseased cells in vitro and in vivo [126].

### 4.4. miR-143 and miR-145

Several studies have found miR-143-3p and miR-145-5p to be dysregulated in both the serum and endometriotic tissue compared to eutopic endometrium and control [105,129,130]. Importantly, miR-143-3p expression was upregulated in the serum of women with endometriosis with a high AUC (0.926), suggesting that miR-143 may a suitable diagnostic biomarker [129]. Single nucleotide polymorphisms with miR-143 have been associated with a higher risk of endometriosis. Allele C rs4705342 and TC genotype rs4705342 with miR-143 was more common in infertile women with endometriosis compared to healthy subjects [131]. The results show increased expression of miR-143 in ectopic and eutopic endometrium compared to normal endometrium [105,130]. A potential target of miR-143 is fibronectin type III domain containing 3B (FNDC3B), which regulates cell motility [100]. It is believed that miR-143 overexpression can inhibit the transcription of FNDC3B by nuclear factor kappa B (NF-kB), and promote cell migration and invasion in endometriosis [130].

Regarding miR-145, its expression in endometriosis is controversial. Cosar et al. (2016) report miR-145-5p to be upregulated in endometriosis, while Wang et al. (2013) reported a decrease in 145-3p expression in the serum of patients [75,129]. The use of different miRNA-145 isoforms in these studies should be emphasized, which may result in different miRNA signatures in the same biological material. Exosomal miR-145 sequencing confirmed that it is involved in immune alteration and cell proliferation, and its expression varies between early and advanced patients with endometriosi compared with controls [98]. In addition, elevated levels of miR-145 in the plasma of patients with endometriosis negatively correlated with the stage of disease [82]. This phenomenon also applies to stemness, where a decrease in relative miR-145 expression in more advanced stages of endometriosis suggests it plays role in the downregulation of pluripotency factors and MSI2 [17,82]. It is postulated that endometriosis is a disease of stem cells because endometrial tissues demonstrate an overall increase in the expression of genes involved in stemness (e.g., *UTF1, TCL1* and *ZFP42*) compared to normal endometrium [82]. miR-145 overexpression has been found to inhibit endometriotic cell proliferation, invasiveness and stemness in an in vitro endometriosis model; this was attributed to regulation of cytoskeletal elements, cell adhesion molecules, protease inhibitors and pluripotency genes (*FASCIN-1*, *JAM-A*, *SOX2*, *SERPINE1*/*PAI-1*, *OCT4*, ACTG2, *TAGLN*, *KLF4*, and *PODXL*) [132]. The expression profile of miR-145 differs between endometrial tissues and blood, and some authors have found it to be upregulated in both eutopic and ectopic tissue compared to healthy endometrium [130]. Others report higher miR-145-5p levels in ectopic endometrial tissue compared to control within the same patient, along with upregulation of VEGFA and markedly decreased levels of epidermal growth factor receptor 2 (EGFR2), phosphatase and tensin homolog (PTEN) and chemokine receptor type 4 (CXCR4) [113]. However, it remains unclear whether the expression of both miR-143 and miR-145 in endometriotic tissue changes with menstrual cycle [130].

### 4.5. Let-7

The let-7 miRNA family consists of eight related sequences (Let-7a/b/c/d/e/f/g/i and miR-98) that regulate a number of basic cell differentiation processes by participating in regulatory loops between downstream genes. The let-7 forms are involved in extensive cellular function modifications and can inhibit cell reprogramming [133]. A relationship has recently been described between reduced levels of several let-7 subtypes both in the circulation of patients with endometriosis, as well as in human endometrial stromal cells, [72,78,134,135]; however, the profile of this miRNAs was dependent on the phase of the menstrual cycle [63]. Significantly lower expression of let-7b, let-7c, let-7d and let-7e was observed in women in the proliferative phase, while in women with endometriosis, higher expression of let-7a, let-7d, let-7e and let-7f was observed in the secretory phase than the proliferative phase, which was not observed in the control group. These findings indicate that circulating miRNA levels do not change in healthy women during the menstrual cycle and are markedly different in the serum of women with endometriosis, highlighting that miRNAs dysregulation is an essential component of the disease [72]. Cho et al. (2015) found a combination of serum let-7b, 7d and 7f levels during the proliferative phase to demonstrate high diagnostic power (AUC = 0.929), suggesting that this panel may serve as a non-invasive diagnostic marker of endometriosis [72]. Moustafa et al. 2020 confirmed significantly lower serum let7b levels in women with endometriosis; this circulating miRNA demonstrated similar value as a diagnostic marker of endometriosis as indicated by Cho et al. 2015 (AUC 0.78 vs. 0.68, respectively). However, unlike Cho 2015, no significant difference in miRNA expression was found between samples collected during the proliferative and secretory phases [72,80]. It is possible that these differences may be due to differences in population type. Indeed, Moustafa et al. 2020 included a range of population types including the Asian population, but the Caucasian population was the most numerous [80]. While Cho et al. 2015, conducted a study only on the Asian population [72]. So far, it has been confirmed that endometriosis is most often diagnosed in Asian populations; however, there is little research in this area and the results are controversial [9]. No comparative studies (meta-analyses) could be found to demonstrate any association between miRNA profile and population type with regard to endometriosis. Interestingly, let7b can differentiate cases of minimal/mild (stage I/II) from controls and also moderate/severe (stage III/IV) endometriosis from controls, but is not useful in distinguishing early from advanced stages of the disease [80]. However, a strong negative correlation has been confirmed between the serum expression levels of let-7b and commonly used diagnostic indices of endometriosis, such as CA-125 [72]. Altered expression of this miRNA has been shown to be a key factor in endometriosis and has a pleiotropic role influencing inflammation, estrogen signaling and growth factor receptors [136]. In addition, other authors have reported that the presence of hereditary polymorphisms in the let-7 microRNA-binding site of the *KRAS* gene leads to abnormal KRAS expression of endometrial stromal cells, as well as increased proliferation and invasion, in women with severe endometriosis [135]. While let-7 down-regulation has been associated with elevated KRAS levels [78,134], an inverse relationship between let-7b expression and the estrogen-regulated gene cyclin D1 has also been documented as a possible mechanism for regulating cell proliferation in endometriosis [72,134].

The importance of the let-7 family in the pathophysiology of endometriosis has also been demonstrated in a mouse model, where the introduction of let-7b-5p locally reduced the level of genes known to influence pathogenesis of endometriosis, such as KRAS4A, KRAS4B, ERα, ERβ, Cyp19a, and IL6 [78,136]. In addition, it was shown that cells treated with a Cyp19a inhibitor had increased levels of let-7f, while the let-7f mimic inhibited Cyp19a expression and reduced cell migration in vitro [134]. Hence, treatments targeting let-7b and let-7f may be a promising therapy for endometriosis. Apart from reducing estrogen signaling (ER and Cyp19A1) and decreasing KRAS, these miRNAs attenuate inflammatory signaling (IL-6), without causing systemic hormonal side effects [136,137]. Transfection experiments have shown remarkable increases in TNF-α, IL-1β, IL-6, and IL-8 in macrophages transfected with a let-7b-5p inhibitor [89]. These factors are upregulated in the peritoneal environment, especially IL6, as endometriotic epithelial cells promote the secretion of IL6 in macrophages [137]. Therefore, it is clear that let-7b plays an important role in macrophage regulation, with a significant decrease in the expression of proinflammatory genes associated with a simultaneous overexpression of the let-7b miRNA and an increase in cytokine expression as a result of let-7b blocking [89] Additionally, down-regulation of let-7b in the endothelium results in higher levels of TGF-β and its receptors, which enhances the EMT process, which plays a key role in the development of endometriosis [73,78,138]. Moreover, down-regulation of let-7 has been shown to correlate with increases in other EMT markers: Twist, Snail 1, vimentin and N-cadherin. In contrast, let-7 overexpression reduces the expression of the mesenchymal markers Snail 1 and N-cadherin, while increasing the epithelial marker E-cadherin [139], which may play an important role in the pathogenesis of endometriosis. There is a need for further experiments to identify the target genes of the let-7 family miRNAs in endometriosis and to test whether modulating these miRNAs has potential therapeutic applications.

## 5. Conclusions

Endometriosis is a common gynecological disease of unknown etiology. The typical clinical symptoms are a serious problem in gynecology and obstetrics, and the disease often remains undiagnosed for several years. Early diagnosis is important in endometriosis, and therefore there is an urgent need to better understand the molecular mechanism underlying the disease progression and to identify novel and more efficient diagnostic predictors for accurate identification of endometriosis, so that optimal therapeutic strategies could be found. There is growing evidence that specific miRNAs are involved in the development and progression of endometriosis through the regulation of broad signaling pathways. Recently, miRNAs have been recognized as promising early biomarkers in endometriosis, because of their tissue-specific expression profiles and noninvasive techniques for diagnosis and prognosis in patients with confirmed diagnosis. While there is agreement among some studies that miRNAs are differentially expressed and the level of their expression varies compared with control tissue, there is also disagreement among these levels of expression. For progress to be made in understanding the role of miRNAs in the pathogenesis of endometriosis, greater emphasis needs to be placed upon standardization of study designs and populations. The significance of EMT as a trigger of epigenetic changes amenable to launch the endometriosis should be also considered.

## Data Availability

Not applicable.
